# Supplementary material for: Equatorial mountains on Pluto are covered by methane frosts resulting from a unique atmospheric process
Source: Nat Commun. 2020 Oct 13;11:5056. doi: 10.1038/s41467-020-18845-3 (PMC7553927; doi:10.1038/s41467-020-18845-3)
Supplement: Supplementary file 1 — Supplementary Information [file 41467_2020_18845_MOESM1_ESM.pdf]

Equatorial mountains on Pluto are covered by methane frosts resulting from a unique atmospheric process

Bertrand et al.

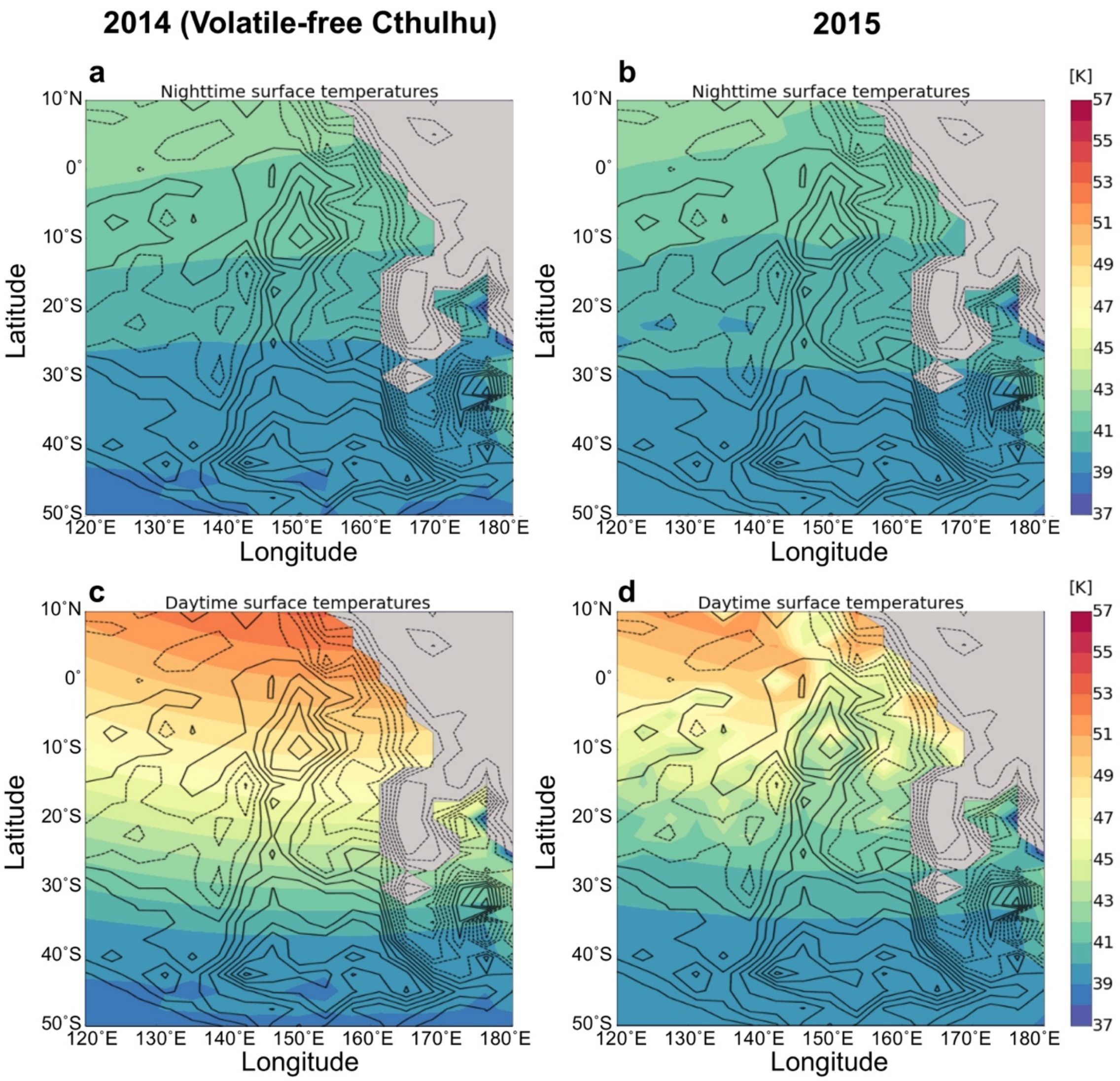

Supplementary Fig. 1. Equatorial surface temperatures on Pluto. Maps of nighttime **(a-b)** and daytime **(c-d)** surface temperatures (K) simulated by the Pluto GCM in Cthulhu in 2014 (when the region is still volatile free, **a-c**) and in 2015 (**b-d**). Daytime surface temperatures in 2015 are decreased on top of Pigafetta Montes due to the formation of bright CH_4 deposits.

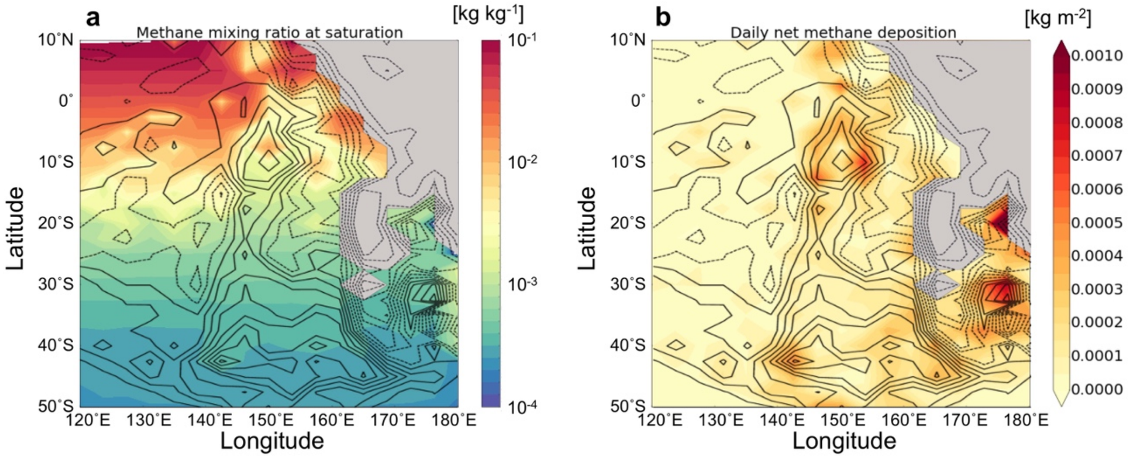

Supplementary Fig. 2. Saturation of gaseous CH_4 above the surface and condensation. (a) Map the CH_4 mass mixing ratio at saturation calculated by the GCM in the region of Cthulhu in 2015. The ratio is lower on top of Pigafetta Montes due to the presence of bright CH_4 deposits and subsequent lower surface temperatures (albedo feedback), which allows for further CH_4 condensation there. (b) Map of the same region showing the daily net CH_4 deposition as obtained with the GCM in 2015. CH_4 mostly deposits on top of the mountain chains, where the nighttime condensation dominates the daytime sublimation, with a net accumulation of ~0.005 kg m^{-2} (or ~1 μm) per Pluto day in July 2015.

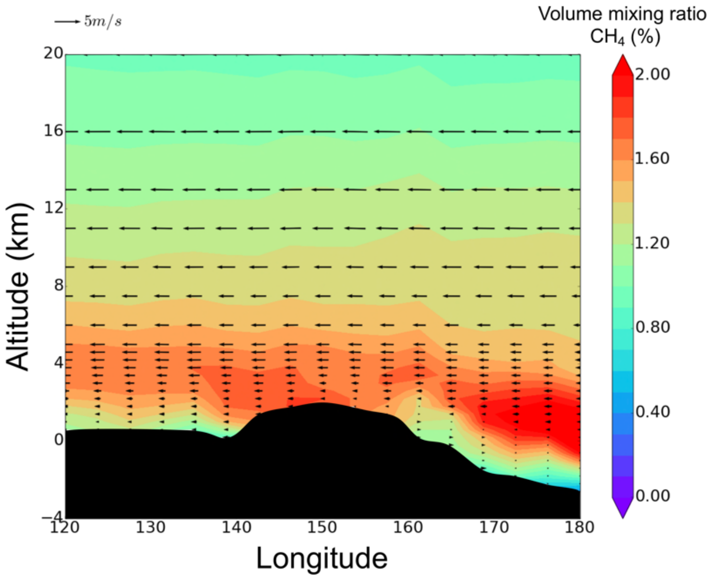

Supplementary Fig. 3. Atmospheric abundance of CH_4 in the model. Cross section at 5°S in the region of Pigafetta Montes showing the volume mixing ratio of methane in the atmosphere as simulated by the GCM in July 2015. An enriched layer of gaseous methane forms at ~4 km altitude. The zonal wind is indicated by the arrows.

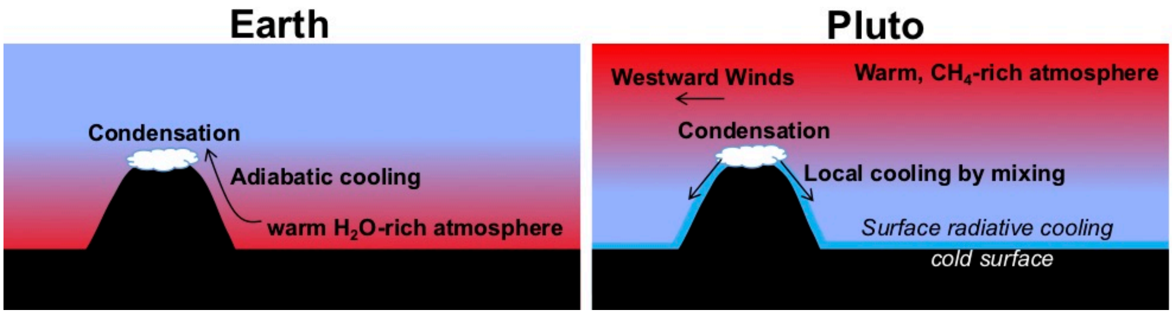

Supplementary Fig. 4. Comparison of the mechanisms forming snow cap mountains on Earth and on Pluto.

Supplementary Discussion

SD1. Equatorial CH_4 frost on Pluto: preference for north-facing slopes and mountain tops.

Supplementary Fig. 5, 6 and 7 show equatorial and mid-latitude regions on Pluto west of Sputnik Planitia where CH_4-rich frosts have been detected by New Horizons' Ralph/MVIC instrument[2,3]. Within the dark terrains of Pluto's equatorial regions, CH_4-rich frost is not only seen at the summit regions of Pigafetta and Elcano Montes, but also on many north-facing slopes located between 20˚S and 10˚N. This includes crater walls and rims (such as Oort, Edgeworth, Brinton and Harrington craters) and the scarps of faults such as Virgil and Beatrice Fossa, as shown by the white arrows in Supplementary Fig. 6.

Farther north in Viking and Venera Terra, CH_4-rich frost is seen to coat the entire rims of impact craters (forming the "bright halo craters"[6]), with more ice occurring on south-facing slopes than north-facing slopes (yellow arrows, Supplementary Fig. 6a-b). Outside Cthulhu, CH_4-rich ice tends to be present in larger amounts at high altitude, as indicated by the 890 nm CH_4 maps of MVIC showing more band depth at altitude[2], even at high latitude. For instance, CH_4-rich ice is seen on the slopes of the mountainous blocks that form Al-Idrisi Montes, at the summits of Tenzing Montes (Supplementary Fig, 6g), and within the high-altitude bladed terrain.

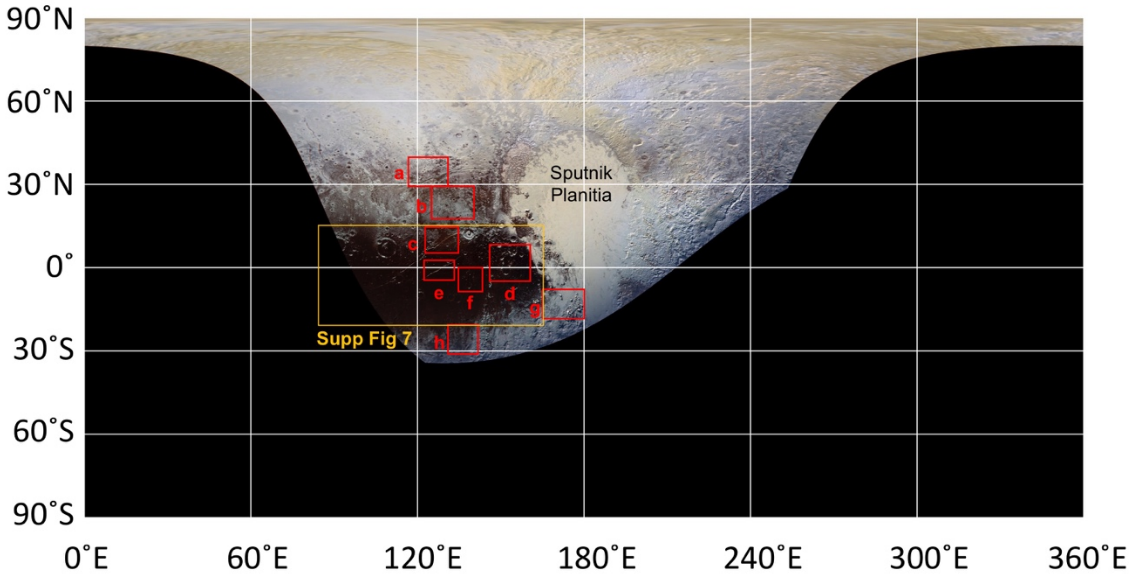

Supplementary Fig. 5. Map of Pluto as seen by New Horizons in July 2015 (cylindrical projections). The red boxes labeled a-f indicate areas shown in detail in Supplementary Fig. 6 and discussed in Section SD1. From North to South: **(a)** Venera Terra and Inanna and Dumuzi Fossae, **(b)** Viking Terra, **(c)** Virgil Fossae, north of Cthulhu, **(d)** Brinton and Harrington craters in Cthulhu, **(e)** Beatrice Fossae in Cthulhu, **(f)** Center Cthulhu, **(g)** Tenzing Montes, south of Sputnik Planitia, **(h)** South Cthulhu. The yellow box indicates the large area of Cthulhu shown in Supplementary Fig. 7.

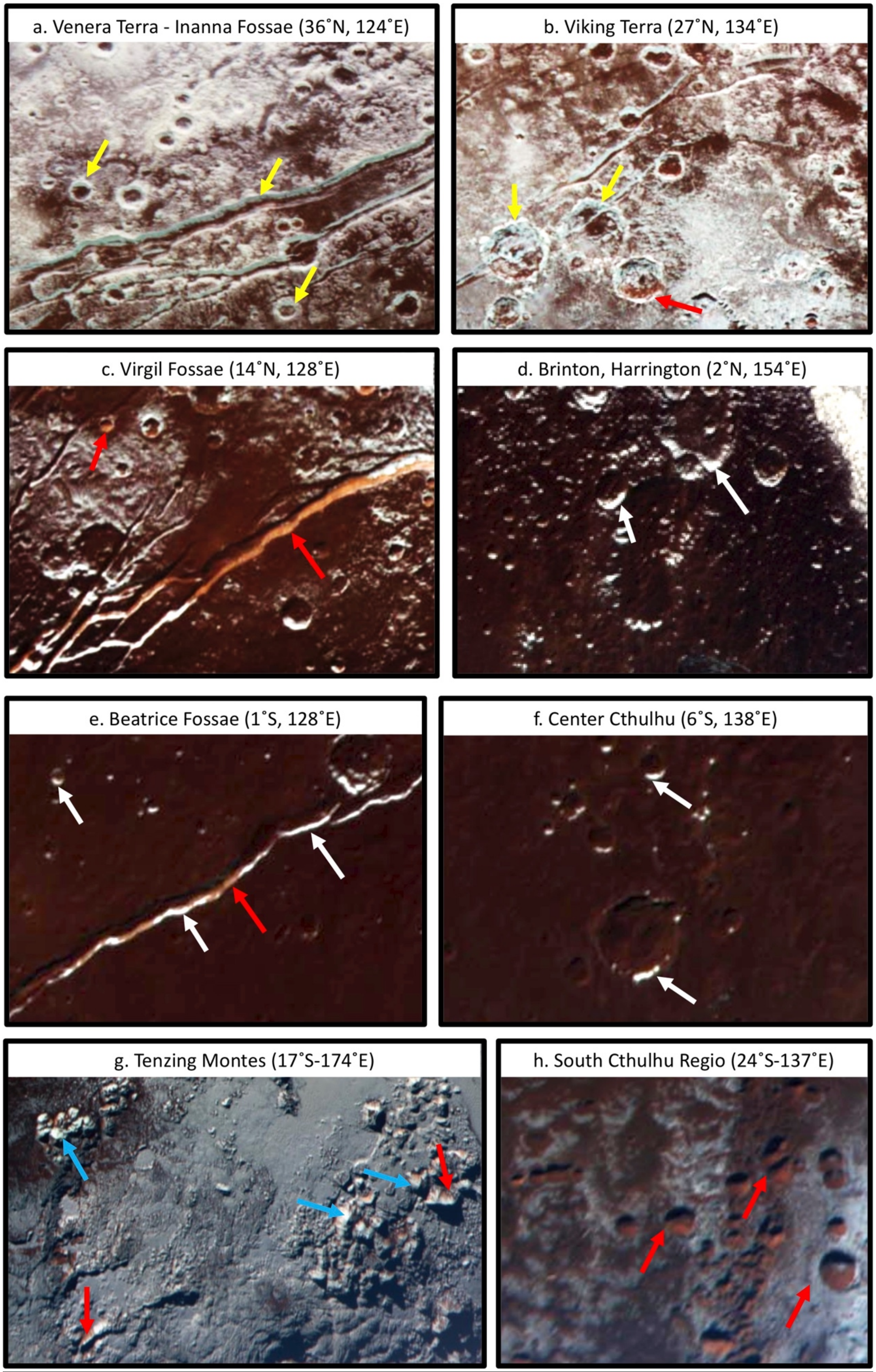
a. Venera Terra - Inanna Fossae (36˚N, 124˚E)
b. Viking Terra (27˚N, 134˚E)
c. Virgil Fossae (14˚N, 128˚E)
d. Brinton, Harrington (2˚N, 154˚E)
e. Beatrice Fossae (1˚S, 128˚E)
f. Center Cthulhu (6˚S, 138˚E)
g. Tenzing Montes (17˚S-174˚E)
h. South Cthulhu Regio (24˚S-137˚E)

Supplementary Fig. 6. CH_4 frost and reddish materials on mountain summits and slopes. Cylindrical projections of Pluto's map showing the locations indicated by the red boxes in Supplementary Fig. 5. Blue arrows show CH_4-ice capped mountains, white and yellow arrows show CH_4 ice on north- and south-facing slopes respectively (craters walls and rims, faults) and red arrows show reddish materials on slopes, that are differentiated from surrounding material of Cthulhu by appearing brighter and redder.

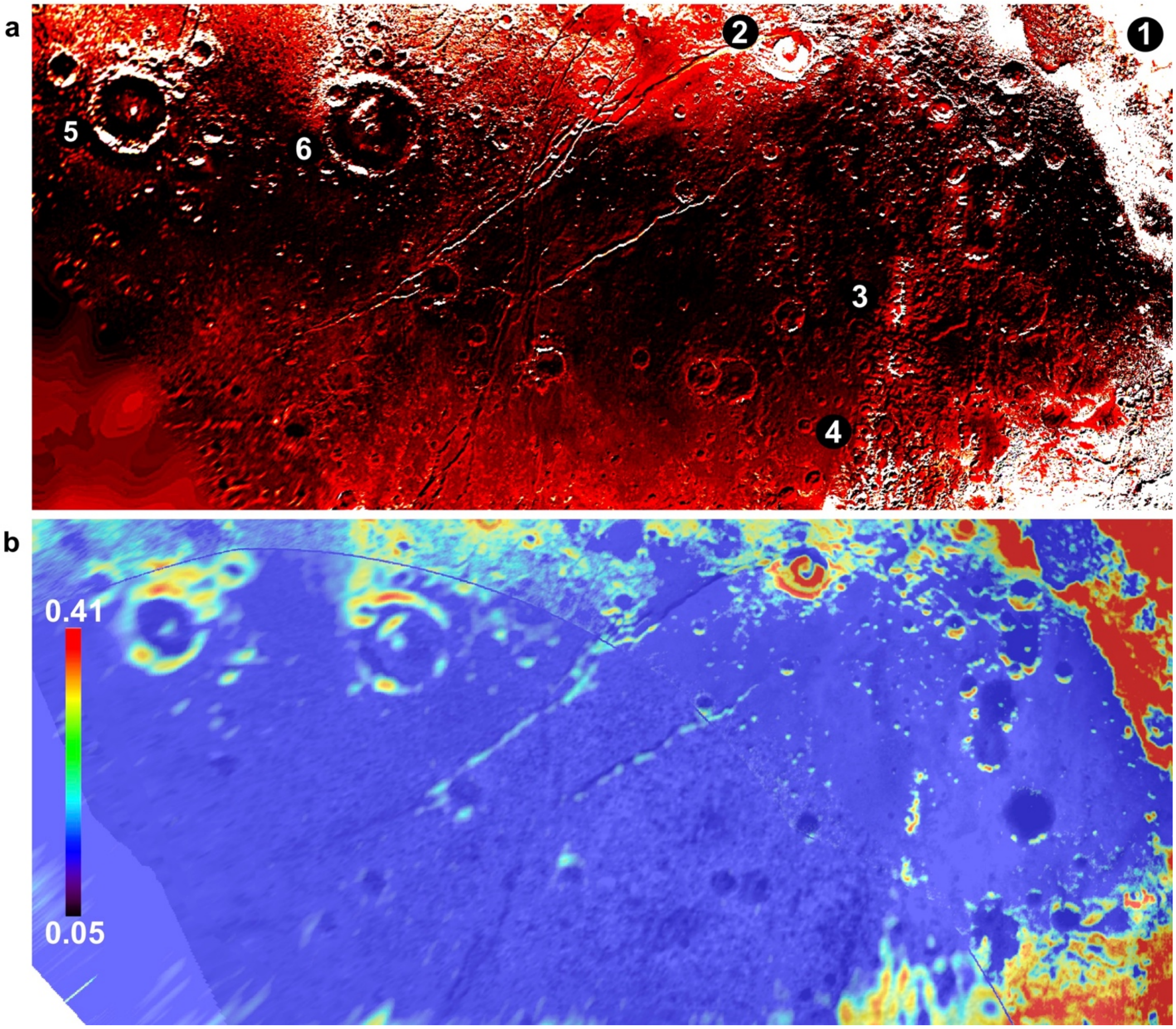

Supplementary Fig. 7. CH_4 frost in the region of Cthulhu. (a) Cthulhu shown in false colours (constructed from blue 400-550 nm, red 540-700 nm, and near-IR filter 780-975 nm images from the Ralph instrument) with a hard contrast stretch to highlight the different colours within the mantling of the materials. The area corresponds to the yellow box in Supplementary Fig. 5. Outside Sputnik Planitia (1) and the floor of Elliot crater (2), which are covered by N_2 ice, the areas in white highlight the presence of fresh, bright CH_4 ice, on top of Pigafetta (3) and Elcano Montes (4), and on the north-facing slopes of many craters in Cthulhu, including the largest craters Oort (5) and Edgeworth (6) in the north-western region of Cthulhu. The red colour is seen in the northern

and southern regions of Cthulhu, but is reddest on many slopes in the centre of Cthulhu, including on crater walls and rims, fault scarps, and at the base of the Pigafetta and Elcano mountains. **(b)** Same region showing the phase index (see definition in "Methods") of CH_4 ice (from blue to red) from the analysis of the LEISA data.

SD2. Investigating the stability of CH_4 frosts on Pluto

In order to explain the CH_4-frosted peaks on Pluto, it has been suggested that the underlying H_2O ice peaks were at some point sufficiently bare to permit deposition of methane such that it could gain at least a seasonally stable foothold[8]. However, our simulations suggest that CH_4-rich ice can deposit onto the dark haze particle blanket in Cthulhu, which despite attaining a higher temperature relative to a higher albedo surface, can become sufficiently cold at night to allow CH_4 to condense. Once established, the higher albedo methane deposit would be colder than the dark surroundings, and so would attract further CH_4 condensation.

In this paper, we highlight two types of CH_4-rich ice deposits observed in Cthulhu, which differ by their mechanism of formation. First, CH_4 ice deposits are observed at high elevation coating the summit regions of mountain ridges (for instance at the summits of Pigafetta Montes). These deposits are the main focus of this study, and we attribute their formation to a local atmospheric-topographic effect leading to an atmosphere enriched in gaseous CH_4 at ~4 km altitude, and thus to saturation in the near-surface atmospheric layer of the mountain's summits. Second, CH_4-rich ice deposits are observed on crater walls and rims, mostly on north-facing slopes in Cthulhu (20°S-10°N). This suggests that the formation of these frosts is controlled by seasonal changes in insolation.

Supplementary Fig. 8 compares the zonal diurnal mean insolation calculated for a flat surface as well as for 30˚ north-facing and south-facing slopes across a Plutonian year and across all latitudes (an inclination of 30˚ is typical of Pluto's topography at km-scale[12]). The season in 2015 was northern spring, which Pluto entered in 1988. In 2015, at all latitudes, the north-facing slopes received more flux than the south-facing slopes (note that the latitudes below 38˚S are in the polar night). However, we hypothesize that the CH_4 ice deposits observed in 2015 by New Horizons on the north-facing slopes of the equatorial regions formed during the immediately preceding northern fall and winter. This period would have commenced in 1840 when the north-facing slopes started to receive less flux than the south-facing slopes, becoming colder and attracting more CH_4 ice as a result. During this period, any CH_4 ice deposits on the south-facing slopes must have sublimed as these slopes received significant insolation (Supplementary Fig. 8d).

Supplementary Fig. 9 compares the zonal mean flux received by flat and 30˚ inclined surfaces, on annual average and on average over the period 1840-2015. On a flat surface, the annual mean flux received is higher at high latitudes than in the equatorial regions, as a result of Pluto's high obliquity. For north-facing slopes, the annual mean flux is lower at southern latitudes and at the equator than it is for northern latitudes, while for south-facing slopes, the flux is lower at northern latitudes and at the equator than it is for southern latitudes. In the equatorial regions, the slopes (e.g. crater walls) receive a lower annual mean flux than the flat surface (e.g. crater floor) does. Since the north- and south-facing walls of equatorial craters receive the same annual mean flux, the CH_4 frosts observed on north-facing walls in 2015 could be seasonal deposits that have been

lingering since the last northern winter, and would presumably disappear over the coming decades as the northern hemisphere advances into summer.

For instance, the CH_4 ice deposits on the north-facing walls may have formed during the period 1840-2015. On average over this period, these slopes receive less flux and are colder than the flat and south-facing slopes (dashed line on Supplementary Fig. 9). The observation of more extended CH_4-rich ice deposits on south-facing slopes above 10˚N cannot be explained by the differences in insolation over this period. Instead, they would be better explained by the annual mean flux.

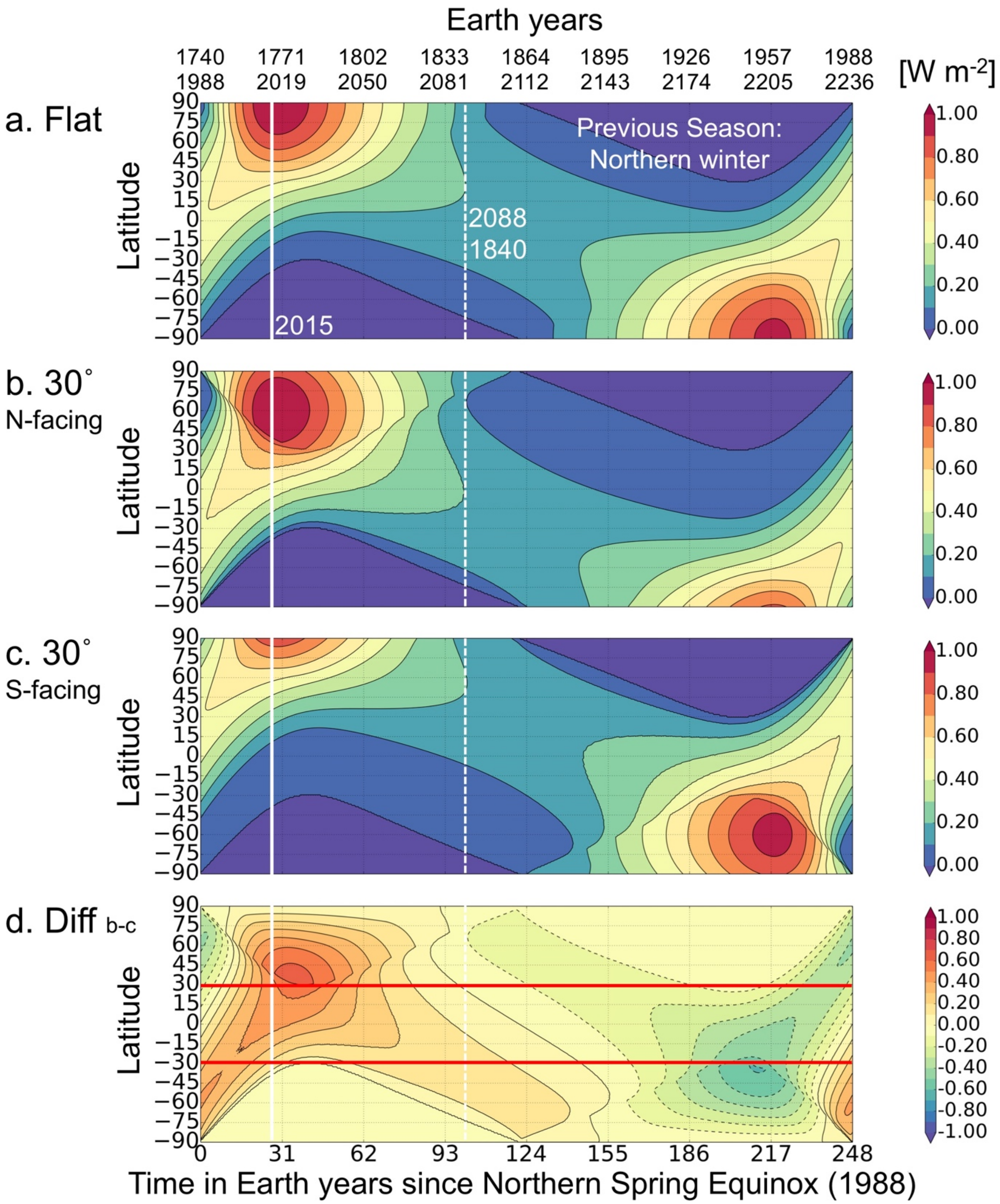

Supplementary Fig. 8. Zonal diurnal mean incident insolation on Pluto over one Plutonian year. **(a)** Received by a flat surface, **(b)** Received by a 30˚ north-facing surface, **(c)** Received by a 30˚ south-facing surface. **(d)** Difference between the zonal diurnal mean insolation received by

a 30˚ north-facing and a 30˚ south-facing surface (**b** minus **c**). The vertical white solid line indicates the year of the New Horizons flyby (2015) and the white dashed line indicates the years 1840 and 2088 (separated by one Plutonian year), which respectively mark the last and the next occasions when the north-facing surfaces start to receive less flux than the south-facing surfaces. The horizontal red lines on **d** indicate the approximate bounding latitudes of Cthulhu (30˚S-30˚N).

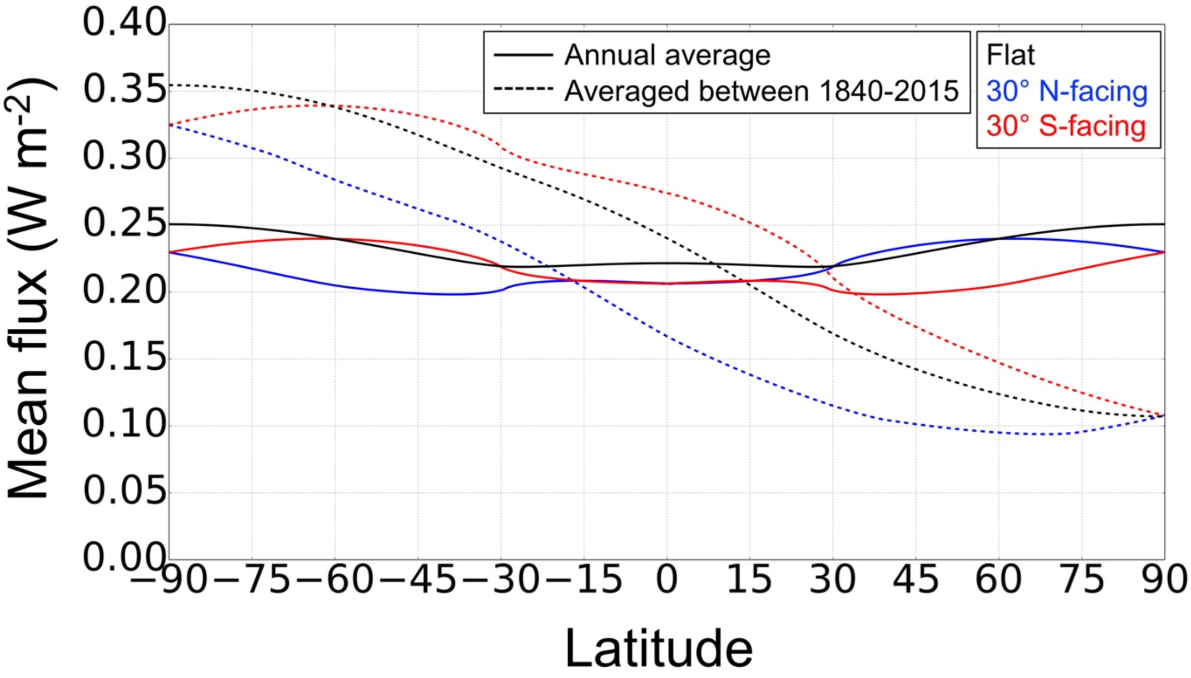

Supplementary Fig. 9. Mean insolation across Pluto's surface. Annual mean insolation (solid lines) and insolation averaged over 175 Earth years (1840-2015, dashed lines) that are received by a flat surface (black line), a 30˚ north-facing (blue line) and a 30˚ south-facing surface (red line).

The reddish material: evidence for past CH_4 frost coverage?

In the southern regions of Cthulhu, between 20˚S and 30˚S, there are craters with no CH_4-rich frost on their north-facing slopes, but which do show a reddish surface (with a higher albedo than the darker plains of Cthulhu around them). Similar reddish areas are seen at the bases of north-facing slopes on mountains in the vicinity of Wright Mons (Supplementary Fig. 6g, while reddish slopes alternate with brighter, frost-covered slopes on the north-facing scarps of Beatrice and Virgil Fossae (red arrows on Supplementary Fig. 6). In general, the northern and southern regions of Cthulhu as well as many crater rims and other peaks within central Cthulhu display brighter and redder brownish colours than the plains of central Cthulhu, as shown by Supplementary Fig. 7a.

Whereas the north-facing scarp of a stretch of Virgil Fossa, with a very distinct reddish colour, may be the signature of cryoclastic deposits[10], the other reddish areas may be indicative of ancient deposition of CH_4 frost that recently sublimed. This hypothesis is supported by the fact that the reddish terrains are observed at similar locations to the CH_4-rich frosts (on the slopes, and in particular on the north-facing slopes in Cthulhu) and sometimes are the extension of CH_4-rich frost patches. For instance, the north-facing scarp of Beatrice Fossa (Supplementary Fig. 6e) shows an alternance of bright CH_4-rich frost and reddish terrain, suggesting that CH_4-rich frosts were covering the entire scarp, with the reddish areas being where portions of the frost cover had recently sublimed. The remaining frost should continue to sublime and disappear, as the incident insolation on these north-facing walls is increasing with time as the northern hemisphere enters summer (Supplementary Fig. ED8b). In the case of Pigafetta Montes, their reddish lower slopes may indicate that the CH_4-rich frost that currently caps them previously extended farther down their slopes. In general, a large area of Cthulhu may have been covered by CH_4-rich frost in a

previous season during which the surface temperatures were colder and the concentration of gaseous CH_4 in the atmosphere allowed deposition at lower elevations.

The locations where the reddish terrains are observed correspond to locations where the water ice bedrock has been detected by LEISA (Supplementary Fig. 7b). This suggests that the deposition and sublimation of CH_4 frost on these slopes have mobilized and depleted dark haze particles on these slopes, thus revealing more of the water ice bedrock.

In this paper, we hypothesize that the CH_4-rich ice seen on top of Pigafetta Montes is seasonal, based on the fact that (1) similar frosts are seen on north-facing slopes but not on south-facing slopes of lower-elevation crater walls/rims and fault scarps in eastern Cthulhu; if the thicknesses of CH_4 deposits on these slopes are the same as on top of Pigafetta Montes, then the CH_4 frosts there may also be seasonal, and (2) brighter and redder brownish colours seem to surround Pigafetta Montes and may be indicative of a more extended CH_4 frost coverage in these regions during a previous season.

However, it remains possible that the CH_4-rich deposits on top of Pigafetta Montes are much thicker than the few millimetres produced by our simulations (maybe up to few metres thick). If so, they could subsist there over multiple Pluto years. A net annual positive mass balance could occur if the amounts of CH_4 involved during the “condensation season” are large enough to subsist during the “sublimation season”. Albedo feedbacks could also help build thicker deposits there beyond a few millimeters.

Would it be possible for climatic patterns across different timescales to ever allow accumulation of the methane deposits onto these mountains to such thicknesses that they might be significant in

terms of affecting the geology and geomorphology of the landscape on which they're emplaced? The thin, localized CH_4 deposits in eastern Cthulhu stand in stark contrast to the expansive CH_4 bladed terrain deposits that form Tartarus Dorsa and cover much of the low-latitudes of the sub-Charon hemisphere, and which reach thicknesses exceeding a kilometre[8,16]. The gradual eastwards tapering of the bladed deposits, such that they vanish entirely at 75°E, forms part of the surficial composition sequence that has been identified within Pluto's equatorial uplands[8], from dominance by nitrogen closest to Sputnik Planitia, to increasing dominance of methane ice to the east culminating in the bladed terrain deposits, to the eventual expiration of large scale deposits of both nitrogen and methane, leaving the landscape of eastern Cthulhu to be mantled by the haze particle blanket, and nitrogen and methane deposits to be limited to small-scale occurrences within deep depressions and mountain peaks/crater rims respectively. This sequence at least partly corresponds to an altitudinal control on ice stability[8], certainly within the localized region covering East Tombaugh Regio and Tartarus Dorsa, but the massive scale of the compositional sequence (covering >300° of longitude from East Tombaugh Regio to eastern Cthulhu) implies that global-scale, longitudinal effects are arguably a more important controlling factor. While it remains to be investigated thoroughly using 3D global climate models, this longitudinal asymmetry has been interpreted as having dynamical origins[18], whereby throughout Pluto's history since the formation of Sputnik Planitia, climatic conditions to the east of Sputnik Planitia have consistently favoured the deposition of CH_4 ice and other volatiles, whereas conditions to the west of Sputnik Planitia have not favoured such deposition. Here, the conditions required for deposition are only met at select localities, including at the summits of high-elevation peaks, or on the pole-facing slopes of craters, meaning that the deposits are limited to being small-scale and transient, and cannot thicken or expand laterally to become geologically significant and perennial units in the way the bladed

terrain deposits have. While it has been observed that the lower slopes of both Pigafetta and Elcano Montes appear dissected, with Pigafetta Montes displaying fluted slopes and Elcano Montes exhibiting dendritic, deeply incised valleys, this morphology has been attributed to glacial erosion by flowing N_2 ice[1], which displays low viscosity at Pluto conditions[2] . CH_4 ice, by contrast, is sufficiently rigid to preclude it from flowing at such conditions[2,3], so the dissected morphology cannot therefore be interpreted as evidence for past, expanded CH_4 ice coverage.

[1] Howard, A. D., and 13 colleagues (2017). Present and past glaciation on Pluto. Icarus 287, 287-300, doi:10.1016/j.icarus.2016.07.006

[2] Eluszkiewicz, J. and Stevenson, D. J. (1990). Rheology of solid methane and nitrogen: applications to Triton. Geophysical Research Letters 17, 1753.

[3] Moore, J. M., and 16 colleagues (2017). Sublimation as a landform-shaping process on Pluto. Icarus 287, 320.
